# Impact of Dietary Patterns on Migraine Management: Mechanisms of Action and Recent Literature Insights

**DOI:** 10.1002/brb3.70652

**Published:** 2025-07-07

**Authors:** Vahideh Behrouz, Elnaz Hakimi, Elias Mir

**Affiliations:** ^1^ Student Research Committee Kerman University of Medical Sciences Kerman Iran; ^2^ Department of Nutrition, Faculty of Public Health Kerman University of Medical Sciences Kerman Iran; ^3^ Department of Clinical Nutrition and Dietetics, Faculty of Nutrition and Food Technology, National Nutrition and Food Technology Research Institute Shahid Beheshti University of Medical Sciences Tehran Iran

**Keywords:** DASH, diet, headache, Mediterranean, migraine

## Abstract

**Purpose:**

Migraine headaches are a common and debilitating neurological disorder that significantly impacts the quality of life of affected individuals. Dietary patterns have been increasingly recognized as potential factors in the prevention and management of migraines. This comprehensive review aims to provide a comparative analysis of various dietary patterns, including the Mediterranean diet (MedDiet), Dietary Approach to Stop Hypertension (DASH), Mediterranean‐DASH Intervention for Neurogenerative Delay (MIND), ketogenic diet, low‐fat diet, low‐glycemic diet, gluten‐free diet, and fasting diet, in their efficacy for preventing and managing migraine headaches, as well as to highlight the underlying mechanisms.

**Methods:**

In this systematic review, PubMed, Web of Science, and Scopus databases were searched from inception to August 2023. Observational and intervention studies were included.

**Findings:**

According to current results, following certain patterns, such as the MedDiet, DASH, MIND, ketogenic, low‐fat diet, low‐glycemic diet, and gluten‐free diet, especially in gluten‐sensitive people, has shown promising results for improving migraine symptoms. Based on the findings, more consumption of plant‐based polyphenols, vegetables, dietary fibers, oily fish, legumes, and low‐fat dairy products—all popular elements of plant‐based dietary patterns like the MedDiet, DASH, and MIND—has been related to improved migraine symptoms. Furthermore, the clinical features of migraine headaches appear to improve with a change in the type and amount of dietary fats. Both the ketogenic diet and the low‐fat diet with an emphasis on omega‐3 fatty acid consumption have provided evidence of promising interventions in the clinical features of migraine headaches. However, fasting or skipping meals aggravates migraine attacks. Overall, these dietary approaches might play a main role in different cellular pathways, including improving mitochondrial function, neuroprotection, regulating vascular tone, inhibiting oxidative stress and neuroinflammation, and reducing levels of calcitonin gene‐related peptides that are involved in the pathogenesis of migraine.

**Conclusions:**

In conclusion, adopting specific dietary strategies may offer a viable approach for individuals suffering from migraines, warranting further research to establish definitive guidelines.

## Graphical abstract

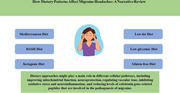

**Abbreviations**
CGRP: calcitonin gene‐related peptides
DASH: Dietary Approaches to Stop Hypertension
DHA: docosahexaenoic acid
EPA: eicosapentaenoic acid,
FFQ: Food frequency questionnaire
HEI: Healthy Eating Index
IL‐1β: interleukin‐1β
IL‐6: interleukin‐6
IκB: inhibitor of kappa B
KD: ketogenic diet
MedDiet: Mediterranean diet
MIND: Mediterranean‐DASH intervention for neurodegenerative delay
MMD: modified MedDiet
NF‐κB: nuclear factor‐κB
NO: nitric oxide
PPAR‐ϒ: peroxisome proliferator‐activated receptor‐ϒ
SCFAs: short‐chain fatty acids
TLR: toll‐like receptor
TNF‐α: tumor necrosis factor‐α

## 1 Introduction

Migraine is a prevalent and chronic neurological condition postulated to originate from a combination of autonomic alterations and neural components (Togha et al. 2021). It is often characterized by a variety of clinical presentations, such as recurrent, throbbing, often unilateral, severe, and pulsating headaches; phonophobia; photophobia; vomiting; and nausea. Some patients may describe transient visual disturbances, including blurry vision, zigzag lines, or flashing lights either before or during an attack (Walter 2022). Migraine is categorized into two primary subtypes: migraine without aura (MwoA) and migraine with aura (MwA). The two types can be clinically differentiated based on the presence or absence of aura symptoms preceding the pain phase. Aura symptoms manifest concurrently with a decrease in regional cerebral blood flow, while in the case of MwoA, regional cerebral blood flow remains within normal limits (Russell et al. 1996). Typical MwA is marked by fully reversible visual and somatosensory manifestations, along with disruptions in speech (Dimitrova et al. 2013).

Migraine directly influences over 1 billion individuals globally across all geographical regions and poses a serious burden on individuals and society (Ashina et al. 2021). It is a multifaceted and intricate condition that is shaped by both genetic predispositions and environmental influences (Gazerani 2020). Although various elements are involved in its pathogenesis, the primary source of the condition remains inadequately elucidated. The mechanisms involved encompass neural damage within the brain, modifications in cerebral blood circulation, neuroinflammatory processes, mitochondrial impairment, hormonal fluctuations, elevated homocysteine levels, obesity, and genetic predispositions. Furthermore, additional contributing factors consist of increased concentrations of specific neuropeptides, particularly calcitonin gene‐related peptides (CGRPs) and nitric oxide (NO), deficiencies in vitamin D, serotonin synthesis by platelets, disruptions in the gut–brain axis, and enhanced production of norepinephrine (Arzani et al. 2020; Dodick 2018; Mottaghi et al. 2013; Oterino et al. 2010). An essential genetic factor may also contribute significantly to the occurrence of migraines. Kowalska et al. emphasize the significant involvement of various ion channels, particularly those within the transient receptor potential ankyrin 1 (TRPA1) family, in the pathophysiology of migraine. The TRPA1 channel is notably responsive to a range of exogenous irritants, which can activate nociceptive pathways that are integral to headache mechanisms. This activation plays a pivotal role in the sensitization of trigeminal neurons, a fundamental characteristic in the onset of migraine episodes. Moreover, other genetic factors linked to migraine, such as mutations in the *SCN1A* and *KCNK18* genes, are also associated with channelopathies relevant to migraine. The discovery of these genetic variants sheds light on the potential mechanisms that contribute to migraine susceptibility and underscores the intricate nature of its genetic framework (Kowalska et al. 2020). Furthermore, migraine has been linked to various other health issues such as obesity, cardiovascular events, respiratory conditions like asthma, and mental health disorders like anxiety and depression (Mousavi et al. 2021). This constellation of comorbidities complicates the clinical management and the outcomes of primary headaches, especially in chronic forms, where symptoms overlap. The mechanisms through which conditions become comorbid remain challenging to elucidate. Comorbidity may serve as a risk factor for the persistence of chronic conditions or act as a precipitating factor for headaches. Additionally, comorbidity could arise as a result of recurrent headache episodes, the effects of headache treatments, or as a consequence of other shared factors associated with headaches (Caponnetto et al. 2021).

Despite evidence that individual food components and nutrients have been related to migraine, there is a limited number of studies examining the correlation between migraine and dietary patterns (Hauge et al. 2011; Kelman 2007; Ulrich et al. 2000). The interaction among foods complicates the study of isolated food components and nutrients in depth. Therefore, it appears that investigating the interrelationships, interactions, and cumulative effects of different nutrients and food items may be more appropriate to examine the relationship between chronic disorders and diet (Hajjarzadeh et al. 2020; Jalilvand et al. 2020; Kant 2004; Karimzadeh et al. 2022). Recent studies have focused on the analysis of dietary patterns to explore the association between dietary intake and the occurrence of disorders (Hu 2002; Hu et al. 1999; Jafari and Behrouz 2023). In this regard, Rist et al. (2015) found that the frequency of migraine attacks varies across different dietary patterns in women with migraine. Another cross‐sectional research indicated that a greater dietary acid load score is inversely linked to migraine headache features among women (Askarpour et al. 2021).

According to our knowledge, few review studies evaluate the correlation between different food patterns and migraine headaches. In this review research, in addition to investigating the effects of different dietary patterns on migraine headaches, the focus has been on the mechanism of action of the constituent elements of these patterns. This is the first time that the therapeutic effects of dietary patterns on various migraine cases, based on the precise mechanisms of the constituent elements, have been examined, and this is a new and important point in the scientific literature in this field. This article, as a comprehensive review of scientific information, provides complementary and valuable roles in scrutinizing the effects of dietary patterns on migraine headaches and contributes to improving attitudes and approaches in the field of nutrition and brain health. So, this narrative review summarizes the available evidence supporting the effects of various food patterns on migraine attacks and discusses the mechanisms of action of these food patterns on migraine headache characteristics among patients with migraine.

## 2 Method

A thorough exploration was conducted until August 2023 utilizing Medline/PubMed, Scopus, and Web of Science to identify intervention and observational studies elucidating the impact of various dietary patterns on migraine headaches. The search concentrated on the subsequent phrases: (“migraine” OR “headache”) AND (“dietary patterns” OR “dietary recommendation” OR “diet” OR “Mediterranean” OR “DASH” OR “low‐fat” OR “gluten‐free” OR “ketogenic” OR “vegan” OR “fasting” OR Diet* guideline* OR eat* pattern* OR Diet* pattern* OR Diet* adhere* OR “gluten‐free” OR “food pattern” OR “low‐glycemic”). The search was limited to publications written in the English language and encompassed all years within the database. Additionally, reference lists of the publications retrieved were scrutinized manually in search of pertinent articles. The selection of relevant articles was contingent upon the assessment of the abstract.

The inclusion of studies was dependent on their provision of information on the relationship (in observational studies) or impact (in intervention studies) of food patterns (as the exposure) on migraine (as the outcome).

The criteria for inclusion involved (a) evaluation of different dietary patterns as the primary exposure; (b) research focused on migraine (including chronic, episodic, MwoA, and MwA); (c) inclusion of people in adulthood and old age (> 18 years); (d) specification of migraine duration for each study (time since migraine diagnosis > 3 months); (e) documentation of the pharmacotherapy used to treat migraine in each study; (f) analytical epidemiological studies, specifically observational research and interventional studies; and (g) publications in English from reputable journals.

The exclusion criteria entailed the absence of original research, such as review articles, case series, studies in animals, and studies involving individuals below 18 years of age.

Identified publications were brought into EndNote referencing software (version X8, Thomson Reuters) for analysis, with titles and abstracts being independently assessed by two researchers (E.H. and V.B.). Full‐text articles were obtained for pertinent records. Any discrepancies were addressed and resolved by consensus among the researchers.

### 2.1 Overview of Dietary Pattern Analysis

Determining food patterns or indexes relies on understanding individuals' total usual consumption of foods, beverages, nutrients, or a combination thereof. Dietary patterns consider the interactive and cumulative impacts between multiple dietary components, reflecting the complexity and totality of real‐world eating behaviors. Food intake data are obtained from interviewer–administered, self‐reported questionnaires, dietary records, or recalls. Most epidemiological research on food patterns has relied on self‐reported food frequency questionnaires (FFQs) to evaluate participants' usual past diets. These questionnaires can include almost 60–200 drink or food items along with portion sizes. Dietary assessment methods are associated with measurement error, which has been discussed in many studies. However, the capacity to rank subjects according to their relative intake of dietary items using the FFQs is commonly considered acceptable (Freedman et al. 2018; Hébert et al. 2014; Steck and Murphy 2020; Subar et al. 2015).

Several advances have occurred in producing different types of food pattern analysis. There exist two primary methodologies for conducting dietary pattern analysis, which hinges on whether the dietary pattern is empirically defined or based on predetermined criteria established by the researcher (Agnoli et al. 2019). An a priori dietary pattern is constructed upon different nutritional variables that can be scored according to dietary guidelines, established scientific correlations between diet and health conditions, or a food tradition. Examples of a priori dietary patterns are the Mediterranean Diet (MedDiet), Healthy Eating Index (HEI), and Dietary Approaches to Stop Hypertension (DASH) score. Their advantages are ease of calculation and interpretation. Limitations include subjectivity in choosing dietary components and may not fully encompass the diet (Agnoli et al. 2019; Hu 2002; Kennedy et al. 1995).

An a posteriori dietary pattern is empirically extracted from data using mathematical and statistical methodologies such as factor analysis or cluster analysis. Its benefits are patterns that reflect how people eat and discoveries that are not limited by current knowledge. Limitations in analysis choices are subjective, and patterns may not be reproducible across populations (Imamura and Jacques 2011; Moeller et al. 2007).

Overall, food pattern analysis is a beneficial strategy in nutrition research to assess the complex synergistic impacts of whole diets on health and disease outcomes. However, traditional single‐food and nutrient analyses are still essential and complementary (Agnoli et al. 2019).

### 2.2 Effects of Various Types of Dietary Patterns on Migraine Headache

In the following paragraphs, we reviewed several dietary patterns and their effectiveness on migraine headaches. The key features of some dietary patterns are described in Table 1. Overall, there is limited research documenting the effects of different dietary factors such as diet quality, macronutrient and micronutrient intake, regularity of meal programs, or different nutritional behaviors and practices in people with migraine. Tables 2 and 3 present studies that have examined the impacts of different dietary patterns on people with migraine.

**TABLE 1:** General characteristics of dietary patterns investigated in migraine headaches.

Dietary pattern	characteristics
Mediterranean diet	Emphasizes plant‐based food items, including whole grains, vegetables, fruits, nuts/seeds, legumes, high consumption of monounsaturated fats (olive oil as the main source of dietary fat), moderate intake of poultry and fish, a moderate alcohol consumption, and low consumption of meat, dairy products, and sweets.
DASH diet	Emphasizes increased intake of whole grains, vegetables, fruits, low‐fat dairy products, nuts, legumes, fish, and poultry. Reduced consumption of red meat, saturated fat, sodium, and sweets.
MIND diet	Emphasizes plant‐based food and encourages a high intake of whole grains, beans, nuts, green leafy vegetables, and berries.
Ketogenic diet	Characterized by very high dietary fat content, moderate protein consumption, and low carbohydrate consumption.
Low‐fat diet	Characterized by the consumption of dietary fats as low as 15%–20% of daily calories.
Low glycemic diet	Emphasizes the selection of foods with a low glycemic index and foods with less glycemic load.
Gluten‐free diet	Characterized by the complete absence of gluten, which is found in wheat, barley, and rye. Carbohydrates are obtained from foods such as rice and corn, which are naturally gluten‐free.
Fasting diet	Characterized as a time‐based energy restriction diet in which eating is prohibited for a limited period.

**TABLE 2:** Observational studies examined the association between dietary patterns and migraine headaches.

First author	Country	Study design	Sample size	Migraine type	Medications	Exposure assessment tools	Type of dietary pattern	Outcomes
Arab et al. (2023)	Iran	Cross‐sectional	*n* = 262 Age = 20–50 years	Migraine (chronic, episodic, MwoA, MwA)	Yes	168‐item FFQ	Mediterranean diet	The highest category of the MD was related to lower headache frequency, headache duration, MHIS, and HIT‐6.
Hande Bakırhan et al. (2022)	Turkey	Cross‐sectional	*n* = 80 Age = 19–64 years	Episodic migraine (MwoA, MwA)	Yes	3‐day food intake records	Mediterranean diet DASH diet	Low adherence to the MD was related to more frequent and more severe attacks. Low adherence to the DASH was related to more frequent and more severe attacks.
Hajjarzadeh et al. (2024)	Iran	Cross‐sectional	*n* = 285 Age = 25–55 years	Chronic migraine (MwoA, MwA)	Yes	FFQ	DASH diet	A higher DASH score was related to a lower frequency and migraine index score in patients suffering from migraine.
Mirzababaei et al. (2020)	Iran	Cross‐sectional	*n* = 266 Age = 18–50 years	Episodic migraine (MwoA, MwA)	Yes	FFQ	DASH diet	Higher adherence to the DASH diet was related to the lower severity and duration of migraine attacks.
Askarpour et al. (2020)	Iran	Cross‐sectional	*n* = 266 Age = 18–50 years	Migraine (chronic, episodic, MwoA, MwA)	Yes	147‐item FFQ	MIND diet	Higher adherence to the MIND diet was related to the lower severity of pain in patients with migraine. There was a negative association between the MIND diet and the duration and frequency of migraine attacks.
Di Lorenzo et al. (2013)	Italy	Prospective observational study	*n* = 108 Age = 19–54 years	Episodic migraine (MwoA, MwA)	Yes	Food records	Ketogenic diet	Headache frequency and drug consumption were reduced in the ketogenic group compared to the low‐calorie diet.
Di Lorenzo et al. (2015)	Italy	Prospective observational study	*n* = 96 Age = 18–65 years	Migraine (chronic, episodic, MwoA, MwA)	Yes	Food records	A very‐low‐calorie ketogenic diet	The frequency of attacks, the number of days with headaches, and medication use significantly reduced after the first month of diet.
Valente et al. (2022)	Italy	Retrospective observational study	*n* = 23 Age = 18–65 years	Migraine (chronic, episodic, MwoA, MwA)	Yes	Food records	Ketogenic diet	The ketogenic diet significantly reduced the number of days with headaches and medication use.
Schröder et al. (2022)	Germany	Retrospective cohort study	*n* = 40 Age = 18–50 years	Migraine (chronic, episodic, MwoA, MwA)	Yes	Digital food questionnaire	Low‐glycemic diet	A low‐glycemic diet significantly reduced migraine frequency and related migraine symptoms.
Lelleck et al. (2022)	Germany	Prospective cohort study	*n* = 71 Age = 18–65 years	Episodic migraine (MwoA, MwA)	Yes	Digital food questionnaire	Low‐glycemic diet	A low‐glycemic diet significantly reduced the number of migraine days by 2.40 days, HIT‐6 improved by 3.17 points, and MIDAS by 13.45 points.
Ameghino et al. (2019)	Argentina	Prospective observational study	*n* = 866 Age = 18–55 years	Migraine (chronic, episodic, MwoA, MwA)	Yes	Food record	Gluten‐free diet	A gluten‐free diet significantly reduced the frequency and intensity of headaches in patients with celiac disease.
Ragab et al. (2021)	Egypt	Prospective cohort study	*n* = 292 Age = 18–55 years	Migraine (chronic, episodic, MwoA, MwA)	Yes	Food record	Ramadan fasting diet	Ramadan fasting significantly increased the frequency of attacks and the use of medication in patients with migraine.
Abu‐Salameh et al. (2010)	Israel	Prospective cohort crossover study	*n* = 32 Age = 18–52 years	Episodic migraine (MwoA, MwA)	Yes	Food record	Ramadan fasting diet	Ramadan fasting significantly led to an increase in the duration of migraine and a decrease in the frequency of attacks in patients with migraine.
Yadav et al. (2010)	India	Prospective cohort study	*n* = 182 Age = 18–58 years	Migraine (chronic, episodic, MwoA, MwA)	Yes	Food record	Fasting diet	A fasting diet significantly increased the clinical characteristics of migraine.
Al‐Shimmery (2010)	Iraq	Prospective study	*n* = 200 Age = 30–39 years	Migraine (chronic, episodic, MwoA, MwA)	Yes	Food record	Ramadan fasting	Ramadan fasting is significantly associated with migraine attacks.

**TABLE 3:** Intervention studies examined the effects of dietary patterns on migraine headaches.

First author	Country	Study design	Duration	Intervention and control groups (*n*)	Treatment	Outcomes
Marchetti et al. (2022)	Italy	RCT	6 weeks	Group A: Modified Mediterranean Diet (*n* = 43) Group B: Modified Mediterranean Diet (*n* = 41)	Group A: Modified Mediterranean Diet with 1.5:1 ω‐6/ω‐3 ratio. Group B: Modified Mediterranean Diet with 4:1 ω‐6/ω‐3 ratio.	Group A showed a significant reduction in both Headache Impact Test‐6 and Visual Analog Scale scores.
Arab, Khorvash, Karimi, et al. (2022)	Iran	RCT	12 weeks	Group A: DASH Diet (*n* = 51) Group B: Control (*n* = 51)	Group A: DASH Diet Group B: Usual Dietary Advice	The DASH diet significantly reduced NO, TOS, and OSI compared to the control group. The DASH diet significantly reduced the clinical indices of migraine, such as MI, HDR, and MHIS, compared to the control group.
Arab, Khorvash, Kazemi, et al. (2022)	Iran	RCT	12 weeks	Group A: DASH Diet (*n* = 51) Group B: Control (*n* = 51)	Group A: DASH Diet Group B: Usual Dietary Advice	The DASH diet significantly reduced the frequency and severity of migraines compared to the control group. The DASH diet reduced the score of depression and stress compared to the control group.
Amer et al. (2014)	USA	RCT	12 weeks	Group A: DASH Diet (*n* = 390) Group B: Control (*n* = 390)	Group A: DASH diet at high, moderate, and low sodium levels Group B: Control diet at high, moderate, and low sodium levels	The occurrence of headaches did not significantly differ between the DASH and control groups during the low, intermediate, or high sodium diet phases. A reduced sodium intake was associated with a significantly lower risk of headache.
Di Lorenzo, Pinto et al. (2019)	Italy	Open‐label, single‐arm, clinical trial	4 weeks	Ketogenic diet	Normo‐caloric ketogenic diet regimen with a ketogenic ratio of 1.7–2:1.	The ketogenic diet significantly reduced the frequency and duration of migraine attacks.
Bongiovanni et al. (2021)	Italy	Open‐label, single‐arm, clinical trial	12 weeks	Ketogenic diet	A ketogenic diet with a limit of 30 g of carbohydrates/day, 1.3–1.5 g/kg body weight of protein, and 35%–80% of total calories from fat.	The ketogenic diet significantly reduced the duration, intensity of pain, days with symptoms, and medication use after 3 months of intervention.
Di Lorenzo, Pinto et al. (2019)	Italy	Randomized, crossover, clinical trial	4 weeks	Group A: very‐low‐calorie ketogenic diet (*n* = 35) Group B: very‐low‐calorie non‐ketogenic diet (*n* = 35)	Group A: protein (≥ 75 g/day), carbohydrates (30–50 g/day), and fats (20 g/day mainly from olive oil). Group B: protein ≅ 50 g/day, carbohydrate ≥ 70 g/day, and fats (20 g/day mainly from olive oil)	A very‐low‐calorie ketogenic diet significantly reduced days with symptoms and migraine attacks compared to a very low‐calorie non‐ketogenic diet. There were no differences in medication use and BMI between the two groups.
Haslam et al. (2021)	Australia	Randomized, crossover, clinical trial	12 weeks	Group A: ketogenic diet (*n* = 16) Group B: anti‐headache diet (*n* = 16)	Group A: ketogenic diet with a ketogenic ratio of 3:1. Group B: A list of permitted and prohibited foods	There were no significant differences in the frequency, duration, and severity of migraine attacks between the two groups.
Bic et al. (1999)	USA	Quasi‐experimental	12 weeks	Low‐fat diet (*n* = 54)	A low‐fat diet with a limited fat intake of no more than 20 g/day	A low‐fat diet significantly decreased headache frequency, intensity, duration, and medication intake.
Bunner et al. (2014)	USA	Open‐label, randomized, crossover trial	6 weeks	Group A: low‐fat vegan diet (*n* = 21) Group B: control group (*n* = 21)	Group A: Provided prescription, then eliminated trigger food and reintroduced Group B: Capsule containing 10 mcg ALA and 10 mcg Vitamin E	A low‐fat vegan diet significantly decreased headache frequency, intensity, and medication intake.
Ferrara et al. (2015)	Italy	Randomized, crossover trial	12 weeks	Group A: low‐fat diet (*n* = 36) Group B: normal‐fat diet (*n* = 47)	Group A: < 20% total daily energy intake from fat. 77 g protein, 32 g fiber, 330 g (63% total E) CHO. 14% MUFA, < 8% sat fat Group B: 25%–30% energy intake from fat. 75 g protein, 32 g fiber, 307 g (56% total E) CHO. 19% MUFA, < 8% sat fat	A low‐fat diet significantly decreased the number of migraine attacks, as well as the severity of attacks, compared to those on a normal‐fat diet.
Evcili et al. (2018)	Turkey	RCT	12 weeks	Group A: low‐glycemic index diet (*n* = 147) Group B: normal diet (*n* = 147)	Group A: A list of permitted and prohibited foods Group B: normal diet with medication (propranolol, amitriptyline, flunarizine, topiramate)	A low‐glycemic diet decreased the visual analog scale scores compared to those in the medication group after 3 months of intervention.

Abbreviations: ALA, alpha‐linolenic acid; BMI, body mass index; CHO, carbohydrate; HDR, headache diary result; MHIS, migraine headache index score; MI, migraine index; MUFA, monounsaturated fatty acids; NO, nitric oxide; OSI, oxidative stress index; TOS, total oxidative status.

#### 2.2.1 Mediterranean Diet

In 2010, the United Nations Educational, Scientific, and Cultural Organization (UNESCO) identified the MedDiet as an “intangible cultural heritage” of Greece, France, Italy, Spain, and Morocco. This dietary pattern aimed to preserve the characteristics of local diversity and culinary traditions unique to Mediterranean countries to promote health benefits (UNESCO 2008). For the first time, it was introduced as a dietary pattern low in saturated fatty acids that was capable of protecting the cardiovascular system. Later, it was identified as a dietary pattern consisting of foods rich in highly protective nutrients that can prevent several diseases (Bakırhan et al. 2022; Keys et al. 1986). The MedDiet is defined by high consumption of plant‐derived food items, such as fresh fruits, vegetables, legumes, seeds, nuts, whole grains, and olive oils, accompanied by moderate consumption of low‐fat dairy items, fish, and red wine (consumed during main meals), as well as limited intake of red meat and sweets. It provides a combination of healthy and diverse foods and many nutritional benefits, such as minerals, vitamins, fibers, and other protective nutrients and compounds (Mentella et al. 2019).

Extensive research efforts have revealed that the MedDiet exerts beneficial effects on numerous disorders, such as obesity, diabetes, cancer, neurological disorders, and cardiovascular diseases. Several investigations have established a correlation between the MedDiet and the clinical aspects of migraine. In a cross‐sectional study, 262 individuals diagnosed with migraine were investigated to show the relationship between following the MedDiet and migraine symptoms. Enhanced compliance with the MedDiet was related to lower headache duration, frequency, migraine headache index score, and Headache Impact Test‐6 (Arab et al. 2023). Consistently, another study found that patients suffering from episodic migraines with low adherence to the MedDiet had more frequent and more severe migraine attacks. Moreover, a remarkable negative relationship was found between following the MedDiet and attack severity (Bakırhan et al. 2022). Marchetti et al. investigated the effects of modified MedDiet (MMD) with a 1.5:1 ω‐6/ω‐3 ratio to a 4:1 ω‐6/ω‐3 ratio on morning headaches. Patients who received MedDiet with a 1.5:1 ω‐6/ω‐3 ratio for 6 weeks demonstrated a remarkable reduction in both Headache Impact Test‐6 and Visual Analog Scale scores. These findings confirm the beneficial effects of the ω‐3‐enriched MMD diet on morning headaches (Marchetti et al. 2022). The main effects of the MedDiet on migraines and headaches remain unknown. Several recent studies have suggested that the specific dietary components and nutrient content of the MedDiet may be associated with improving brain health. Here, we describe the effects and mechanisms of action of some components of the MedDiet on migraine clinical features.

‐ *Plant polyphenols*: Fresh fruits, vegetables, and olive oil are the major sources of polyphenols in MedDiet, which exert biological properties such as anti‐inflammatory, antioxidant, antimicrobial, and antiviral activities (Pergolizzi et al. 2019). Several studies indicated that plant foods, through their polyphenol content, have positive effects on neuroinflammatory signaling cascades, inducing neuroprotective properties (Baron 2018; R. Huang, Zhu, et al. 2023). These components inhibit the expression of proinflammatory markers, such as cytokines, chemokines, interleukins, and monocyte chemoattractant protein‐1, by downregulating nuclear factor‐κB (NF‐κB), PI3K/AKT, JAK/STAT, and MAPK cascades (Chinta et al. 2013; Morshedzadeh et al. 2023; Subedi et al. 2021; Subedi et al. 2017). Polyphenols can target NF‐κB through toll‐like receptor (TLR) signaling pathways in multiple ways. They modulate the expression of toll‐interacting protein, exert neuroprotective properties through the TLR4/NF‐κB/STAT signaling pathway, and decrease neuronal apoptosis (Rahimifard et al. 2017). Other neuroprotective effects of plant polyphenols include suppression of activation of microglia, astrocytes, and immune cells like mast cells and inflammatory cytokines released from these cells (Kempuraj et al. 2021).
‐ *Omega‐3 fatty acids*: Long‐chain omega‐3 fatty acids, such as eicosapentaenoic acid (EPA, C20:5) and docosahexaenoic acid (DHA, C22:6), derived from seafood and fish, are more frequently ingested in MedDiet, distinguishing it from other dietary patterns (Tangney et al. 2014). Neuroinflammation has a main role in the progression and pathogenesis of migraine headaches, which can sensitize and activate perivascular meningeal afferent nerves (Gerring et al. 2018; Levy 2010). In addition, activated glia leads to the generation of pro‐inflammatory markers that can damage the blood‐brain barrier. Modulation of fatty acids in the microenvironment can alter physiological functions related to cell signaling cascades (Vigh et al. 2005).

**FIGURE 1:**
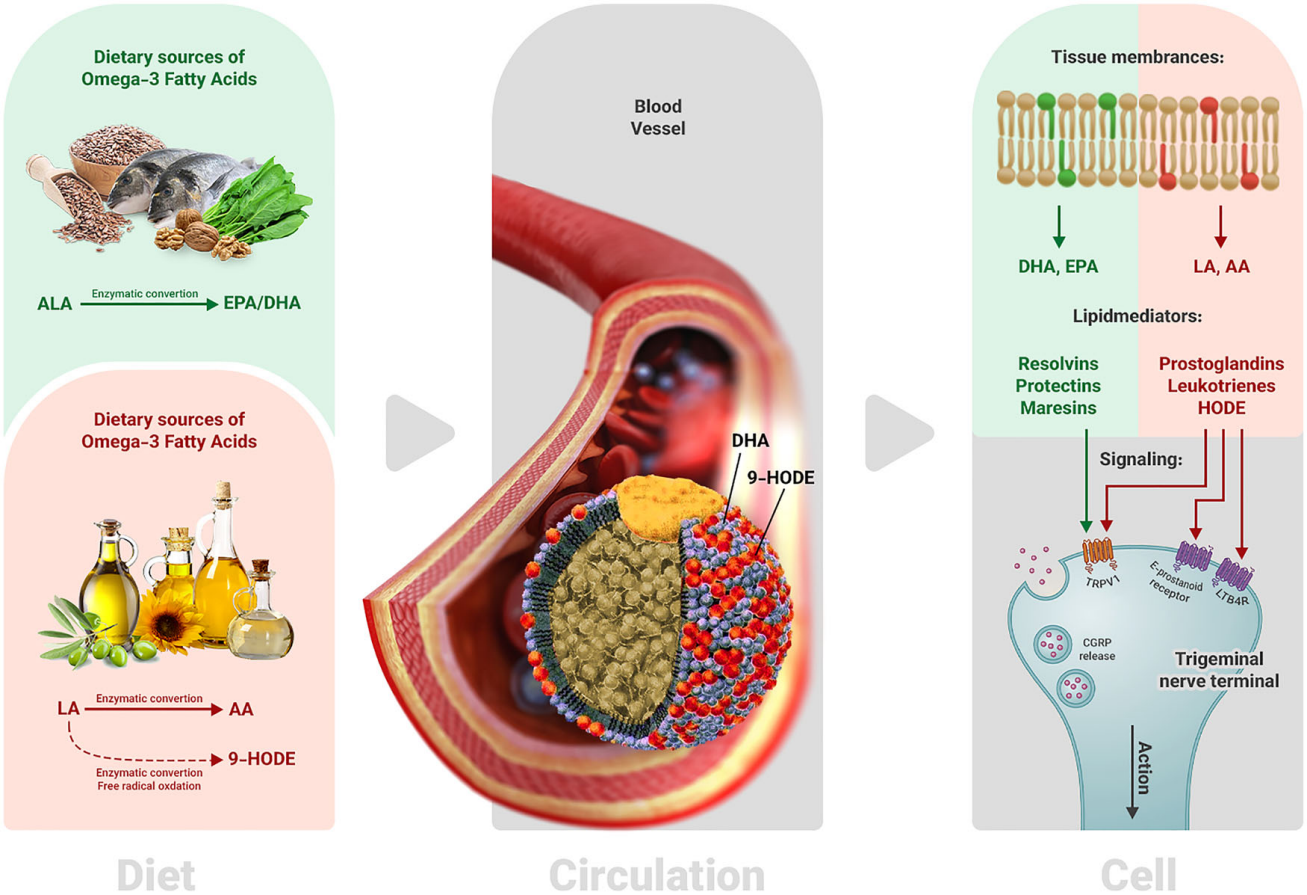
Proposed mechanisms relating dietary sources of omega‐3 and omega‐6 fatty acids to migraine pathogenesis. Diet section: dietary sources of fatty acids endogenously converted into different mediators. Dietary ALA and LA are converted into EPA/DHA and AA/9‐HDOE through an enzymatic reaction, respectively. Circulation section: circulating blood contains a set of omega‐3 and omega‐6 fatty acids. Dietary fatty acids and their mediators are mainly esterified to triglycerides, cholesterol esters, and phospholipid components of lipoproteins. Cell section: omega‐3 and omega‐6 fatty acids are the main components of tissues' lipid membranes that are innervated by the trigeminal nerve and act as precursors for the synthesis of oxylipins. High consumption of dietary omega‐6 fatty acids increases the generation of pro‐nociceptive mediators, such as prostaglandins, leukotrienes, and 9‐HODE. In contrast, high consumption of dietary omega‐3 fatty acids enhances the generation of anti‐nociceptive mediators, such as resolvins, protectins, and maresins. Oxylipins modulate GPCRs and TRP channel receptors for trigeminal nerve activation and local CGRP release. AA, arachidonic acid; ALA, α‐linolenic acid; DHA, docosahexaenoic acid; EPA, eicosapentaenoic acid; HODE, hydroxy octadecadienoic acid; LA, linoleic acid; TRPV1, transient receptor potential vanilloid, type 1.

Omega‐3 fatty acids are considered precursors to various families of bioactive lipid mediators called oxylipins, which have potent analgesic (pain‐reducing) properties (Ramsden et al. 2021). Numerous studies have described the treatment impacts of n‐3 fatty acid or fish oil on migraine features. In a systematic review with meta‐analysis, n‐3 polyunsaturated fatty acids were examined in relation to the frequency, duration, and severity of migraine attacks. The findings demonstrated that n‐3 fatty acids administration did not significantly affect the severity and frequency of migraine but had a remarkable reduction of approximately 3.44 hours in migraine duration (Maghsoumi‐Norouzabad et al. 2018). However, the majority of included articles had methodological restrictions, including limited sample size, inappropriate control group, and some confounding factors. Another research indicated that diets with increased content of n‐3 fatty acids, including EPA and DHA, could reduce both the duration and frequency of migraine headaches (Ramsden et al. 2021). In vitro studies have shown that n‐3 fatty acids, targeting lipopolysaccharide surface receptors, suppress the activation of NF‐κB and the generation of inflammatory mediators in microglia. Moreover, they produce some oxylipins such as resolvin and protectine through the cyclooxygenase and lipoxygenase enzymatic pathways, which induce anti‐inflammatory effects by suppressing the generation of inflammatory markers like tumor necrosis factor‐α (TNF‐α), interleukin‐1β (IL‐1β), and interleukin‐6 (IL‐6) in various cell types like microglial cells, thereby reducing pain (Layé 2010; Soveyd et al. 2017; Xu et al. 2010). The anti‐inflammatory properties of n‐3 fatty acids involve the reduction of MAPK activity, phosphorylation of inhibitor of kappa B (IκB), which decreases the activity of NF‐κB, and interaction with peroxisome proliferator‐activated receptor‐ϒ (PPAR‐ϒ). Omega‐3 fatty acids are known ligands for PPAR‐ϒ, a transcription factor, leading to anti‐inflammatory actions (Soveyd et al. 2017). Oxylipins also directly affect vascular tissues, promoting vasodilation and increasing vascular permeability (Ramsden et al. 2021) (Figure 1).

‐ *Dietary fibers*: The MedDiet is renowned for its elevated consumption of whole grains, vegetables, and fruits, which provide high amounts of dietary fibers. These food groups are reported to be rich in phytochemicals like phenolic acid, phytosterols, and β‐glucans, which are attributed to their considerable antioxidant characteristics (G. Morris et al. 2017). Whole‐grain cereals, vegetables, and fruits represent primary reservoirs of fermentable carbohydrates, encompassing dietary fibers, oligosaccharides, and resistant starch, which are typically indigestible by human intestinal enzymes. However, the intestinal microflora selectively decomposes and ferments them into gases and short‐chain fatty acids (SCFAs) (J. Slavin 2004). SCFAs are assimilated and employed by the gut epithelial cells alongside beneficial bacteria, including *lactobacilli* and *bifidobacterial*, thus amplifying the intestinal microbial ecosystem, reducing the intestinal pH, and improving the body's immune defenses (Gill et al. 2021). Numerous research endeavors have validated that a suitable augmentation in the consumption of food groups providing dietary fibers can foster the growth of probiotics and improve the functions of intestinal and distal organs (Dvoncova et al. 2010; Peterson et al. 1995). On the other hand, emerging evidence supports the involvement of gut microbiota in brain function through the gut–brain axis, thereby regulating neurological functions, including cognition, behavior, and even nociception (Mayer 2011). The gut–brain axis comprises an intricate framework of neural, immunological, and endocrinological factors, serving as a primary target for managing brain health (Kim et al. 2021). The composition of gut microbiota affects the gut‐brain axis through direct vagus nerve stimulation and indirect signaling (hormones, microbiota‐derived molecules, and inflammatory mediators). It is supposed that several neurotransmitters, such as serotonin, dopamine, gamma‐aminobutyric acid, and CGRP, participate in the correlation between the gut and the brain in migraine disease (Arzani et al. 2020). Studies using germ‐free mice show heightened plasma concentrations of serotonin and tryptophan. It has been found that nutritionally manipulating gut microflora can reduce tryptophan availability and serotonin synthesis, influencing susceptibility to migraines (Mittal et al. 2017). CGRP, one of the key biomarkers of migraine, has been implicated in several pathophysiologic processes underlying migraine attacks. It plays a function in inflammation, digestive nociception, motility, and gastric acid secretion (Aurora et al. 2021). Gut microbial dysbiosis with dominant *Escherichia coli* and *Enterococcus faecalis* contributes to altered neurotransmitter release (CGRP, neuropeptide Y, and substance P), which can incite and activate pain receptors that predispose to migraine attacks (Balan et al. 2021). Modulating the gut microbiota by administering probiotics and dietary fibers may reduce migraine attacks by decreasing inflammatory factors and modulating neurotransmitters (Durham 2006).

**FIGURE 2:**
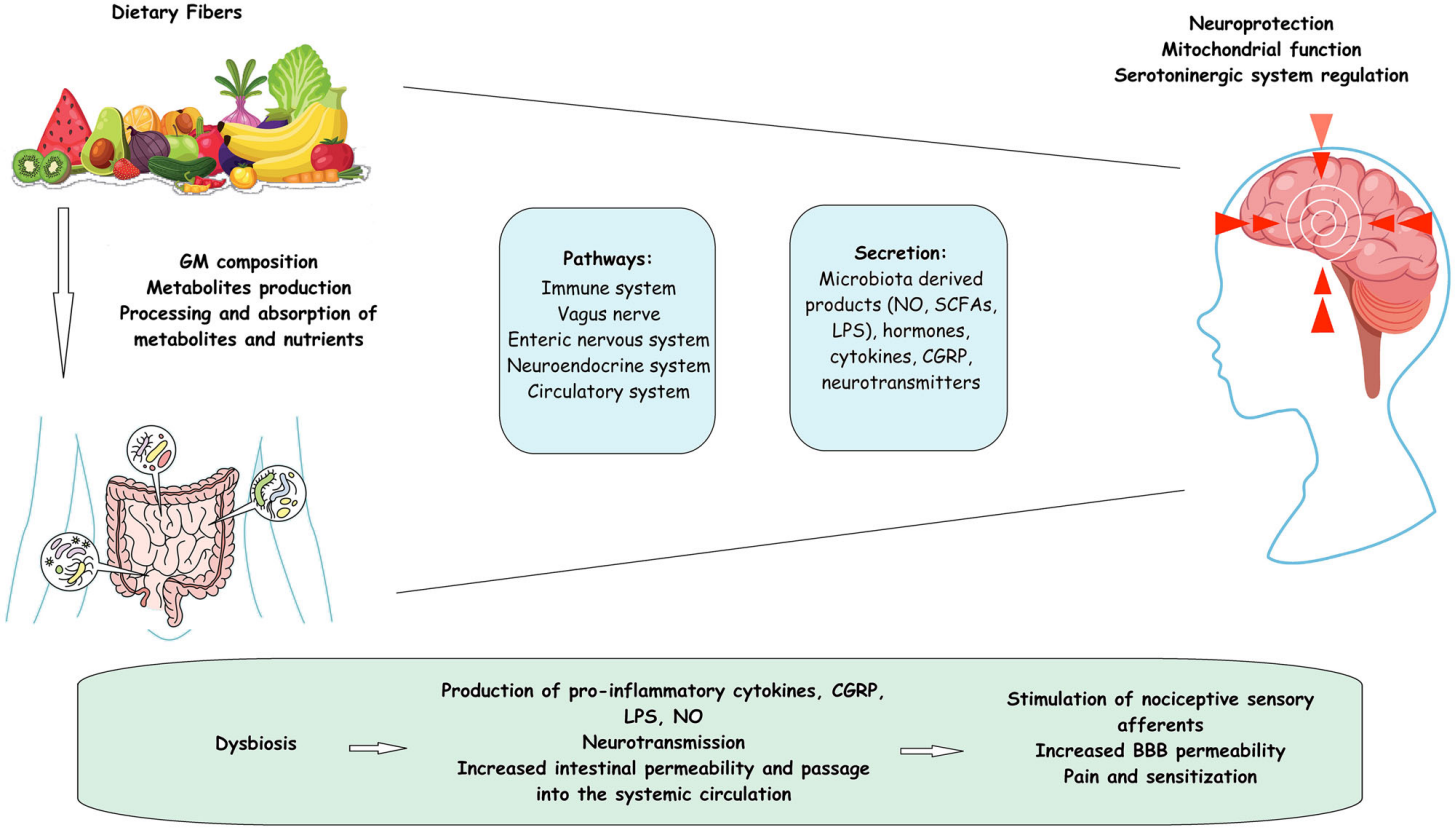
The relationship among dietary fibers, the gut microbiome, and the brain in the development of migraine.

On the other hand, immune cells and inflammatory mediators in the gut, through increased gut permeability, can sensitize trigeminal pain pathways involved in migraine and other afferent nerve endings and induce visceral pain. Nutritional factors like dietary fiber and SCFAs from gut bacteria affect gut and immune function, which can impact brain health (Bruno et al. 2007; Martami et al. 2018). Evidence of the impact of dietary fibers on migraine symptoms is rare. However, a cross‐sectional study that included 12,710 participants suffering from migraines revealed a notable inverse correlation between the intake of dietary fiber and migraines. Specifically, for every 10 g/day augmentation in dietary fiber intake, there was an 11% reduction in the occurrence of migraine headaches (H. Huang and He 2023). However, further studies are required to confirm these observations (Figure 2).

#### 2.2.2 DASH Diet

The DASH diet was developed in the 1990s by the National Heart, Lung, and Blood Institute as a treatment for hypertension. This dietary pattern emphasizes higher intakes of fruits, vegetables, legumes, nuts, and whole grains, alongside advocating for moderate consumption of low‐fat dairy items, decreased consumption of animal proteins, and a controlled sodium intake (Song et al. 2021). The DASH dietary plan is abundant in magnesium, potassium, calcium, and dietary fibers while restricting the intake of total fat, saturated fat, cholesterol, and sweetened beverages. Regarding key micronutrients that play an essential role in cardiometabolic health, it recommends daily intakes of 1250 mg of calcium, 500 mg of magnesium, 4700 mg of potassium, and 2300 mg of sodium (Lari et al. 2021). Subsequent research has increasingly highlighted the neuroprotective properties of the DASH dietary regimen. Several observational studies have scrutinized the association of adherence to the DASH dietary pattern with the pain intensity and frequency of migraine attacks. The findings demonstrated that a higher DASH adherence score correlates with reduced frequency, severity, duration, and migraine index score among migraine sufferers (Hande Bakırhan et al. 2022; Hajjarzadeh et al. 2024; Mirzababaei et al. 2020). In a randomized clinical trial, following the DASH diet for 3 months could reduce some oxidative stress parameters, including NO, total oxidative status, and oxidative stress index in comparison to the control group in individuals with migraine. Moreover, adherence to the DASH diet resulted in a remarkable reduction in the clinical indices of migraine at the end of the intervention (Arab, Khorvash, Karimi, et al. 2022). Similarly, Arab et al., in a randomized clinical trial, showed the impacts of the DASH dietary pattern on mental health, clinical results, and quality of life among women suffering from migraines. The clinical manifestations of migraine (frequency and severity), as well as the quality of life and mental health outcomes (depression and stress), significantly improved in the DASH group compared to the control post‐intervention, supporting the protective role of the DASH dietary plan on migraine health outcomes (Arab, Khorvash, Kazemi, et al. 2022). Given that both the MedDiet and the DASH diet are plant‐based, many of their mechanisms of action concerning migraines exhibit similarities. The specific mechanisms of action of other components of the DASH diet are elucidated below:

‐ *Sodium*: Several investigations have found that an elevated consumption of sodium is related to increased severity and frequency of migraine attacks in some individuals. Although reducing sodium intake is beneficial for older adults, one study in young, nonhypertensive women with a normal or low BMI found that a high‐sodium diet was beneficial in this population (Stanton 2016). This finding suggests that sodium intake in patients with migraine should be tailored to their health condition. Another clinical trial investigated the effects of DASH and a low‐sodium regimen on headaches. Individuals were divided into DASH and control groups in three 30‐day phases (high, moderate, and low sodium diets). The occurrence of headaches did not show significant differences between the DASH and control groups during the high, intermediate, or low sodium diet phases. However, the risk of headache was lower with reduced sodium consumption compared to high sodium intake, regardless of whether participants were following the DASH diet or control diet (Amer et al. 2014). Consistent with Chen et al. (2016), demonstrated that a decrease in sodium consumption could decrease the frequency of headaches in elderly subjects with hypertension. Moreover, a recent observational study was carried out to examine the association between 24‐h urine sodium intake and clinical characteristics of migraine. The urine sodium was positively related to extended duration of migraine attacks and elevated scores on the migraine headache index (Arab, Khorvash, Heidari, et al. 2022). The mechanisms linking dietary sodium intake with migraine are not fully understood. In general, cations play important roles in brain function and are implicated in migraine pathophysiology. Prior studies have found elevated sodium levels in the blood and cerebrospinal fluid during migraine episodes (Campbell et al. 1951; Harrington et al. 2006). This highlights a potentially distinctive role of sodium in migraine. Elevated extracellular sodium can reduce neuronal resting membrane potential and action potential threshold, leading to cerebrovascular reactivity associated with migraine. It can also diminish repetitive firing thresholds by amplifying sodium conductance and enhancing pH–pH‐triggered nociceptor firing (Zhang 2019). Additionally, salt loading may trigger migraines by increasing angiotensin, aldosterone, and NO production, promoting endothelial dysfunction, and upregulating inflammatory pathways (Brainard 1981; Neeb and Reuter 2007; Zhu et al. 2014). In summary, further investigations are imperative to unveil the precise mechanisms connecting sodium intake to migraine pathophysiology.
‐ *Magnesium*: There are several important food sources of magnesium in the DASH dietary pattern, including green vegetables, legumes, whole grains, seeds, and nuts (Duffine and Volpe 2013). Research has demonstrated that individuals suffering from migraines exhibit lower serum magnesium levels compared to those without migraines, with magnesium deficiency strongly linked to migraine occurrences (Silva et al. 2023; Talebi et al. 2011). Recent observational studies have highlighted that a higher dietary magnesium intake is associated with lower odds of migraine attacks compared to the lowest quantile of dietary magnesium intake (M. Slavin et al. 2021). Magnesium plays a crucial role in supporting the optimal operation of the nervous system. It blocks the calcium channel in *N*‐methyl‐d‐aspartate receptors to inhibit the neuronal influx of calcium ions, thereby protecting cells from overexcitation (Domitrz and Cegielska 2022). Moreover, it is known that hypomagnesemia is associated with increased cyclic AMP levels, releasing neurotransmitters responsible for disturbances of vasomotor, which are associated with headaches during migraine attacks (Domitrz and Cegielska 2022). Other mechanisms of action of magnesium in preventing and managing migraine include counteracting vascular spasms, improving mitochondrial oxidative phosphorylation, reducing inflammatory marker production, and enhancing the transmission of serotonin receptors (Meng et al. 2021). In general, dietary patterns with high amounts of magnesium can be beneficial for brain health, neurological functions, and especially migraine management.
‐ *Calcium*: Dairy foods, grains, soy products, and seafood are calcium food sources in the DASH diet. Research exploring the correlation between dietary calcium and migraines remains limited. Within a cross‐sectional study involving 10,798 adults experiencing severe headaches or migraines, it was found that a high dietary consumption of calcium was inversely related to migraines (Meng et al. 2021). Moreover, Silva et al., in observational research, demonstrated that plasma levels of calcium in migraineurs are significantly lower than those of patients without migraine (Silva et al. 2023). Given that dairy items are primary calcium sources, Arianfar et al. investigated the relationship between dairy consumption and migraine among children and teenagers. The study findings showed that increased dairy intake could attenuate the odds of migraine attacks (Ariyanfar et al. 2022). Another study explored the link between dairy consumption and migraines in the adult demographic, with results confirming the negative correlation between dairy intake and migraines. While the precise mechanisms linking dietary calcium and migraines remain unclear, it is suggested that dietary calcium might mitigate various aspects of neuroinflammation and enhance anti‐inflammatory cytokines involved in managing migraine episodes and headaches (Ariyanfar et al. 2022). Further research is imperative to thoroughly investigate how dietary calcium may impact migraine headaches.

#### 2.2.3 Mediterranean‐DASH Intervention for Neurodegenerative Delay Diet

Similar to the MedDiet and the DASH dietary patterns, the Mediterranean‐DASH intervention for neurodegenerative delay (MIND) diet supports natural plant‐based food items with a restriction of high‐fat and animal foods. However, some differences were apparent between the MIND, the DASH, and the MedDiet. The MIND diet, for instance, places importance on a high intake of green leafy vegetables and berries, which is not the case for the DASH and MedDiet. Conversely, the MIND dietary pattern does not specifically address the high intake of dairy, fruits, and potatoes. Another disparity among these dietary patterns pertains to fish consumption recommendations. In the MIND diet, individuals benefit from consuming at least one serving of fish weekly, whereas the MedDiet and DASH require higher fish intake for favorable outcomes (Grajek et al. 2022). Previous studies revealed that the MIND diet is related to neuroprotection and brain health promotion. Higher adherence to this pattern could reduce the rate of cognitive decline, making individuals appear 7.5 years younger (M. C. Morris et al. 2015). However, limited research has assessed the association of the MIND diet with the clinical characteristics of migraines. A cross‐sectional study involving migraine patients revealed that following the MIND dietary pattern led to a notable decrease in migraine‐related disability assessment, frequency, and duration of headaches (Askarpour et al. 2020). Many of the plausible mechanisms underlying the impact of the MIND diet on migraines resemble those of the Mediterranean and DASH diets. As mentioned, increased levels of certain neuropeptides, such as CGRP, through neuroinflammation and dilation of dural and cerebral blood vessels play an important role in migraine pathophysiology. Growing evidence suggests that dietary antioxidants can block the effects of CGRP. Dietary components, including vegetables, fish, plant oils, berries, and nuts, may benefit migraine by reducing oxidative stress and neuroinflammation (Askarpour et al. 2020; Gómez‐Pinilla 2008). However, research is needed to find out more precise mechanisms.

#### 2.2.4 Ketogenic Diet

The ketogenic diet (KD) is a dietary treatment strategy for epilepsy and presents as a promising therapeutic option for various neurological disorders. This dietary pattern is a very low‐carbohydrate (less than 30 g/day or 5% of total calories) dietary approach that is based on the consumption of adequate protein and high amounts of dietary fats, resulting in increased ketone body levels in the cells (Paoli et al. 2021). Hepatocytes predominantly synthesize ketone bodies (acetoacetate, β‐hydroxybutyrate, and acetone) as energy‐dense substrates, particularly beneficial for the brain and heart when dietary glucose is limited or severely restricted. Elevated ketone bodies may affect migraine pathophysiology through several mechanisms. First, these substrates produce lower levels of inflammatory mediators and oxidative stress markers, along with higher levels of antioxidant proteins, including mitochondrial superoxide dismutase, metallothionein, and catalase (Gross et al. 2019). Second, considering the role of mitochondrial functioning in the pathogenesis of migraines, animal model studies have shown that ketone bodies may enhance mitochondrial function and biogenesis by maintaining mitochondrial respiration and ATP production (Bough et al. 2006; Srivastava et al. 2012). Third, it is recognized that ketone bodies can counteract or prevent certain detrimental effects associated with hypoglycemia and/or hypometabolism, which are generally found to correlate with migraine attacks (Gross et al. 2019). Other known mechanisms are that ketosis can lead to migraine relief by compensating for serotonergic disturbances, inhibiting cerebral excitability, reducing the production and release of CGRP, and altering cortical spreading depression (Gross et al. 2019; Razeghi Jahromi et al. 2019).

Evidence is emerging to support the efficacy of ketogenic diets in the treatment of neurological disorders, such as migraine, brain tumors, neurodegenerative disorders, and amyotrophic lateral sclerosis, in addition to refractory epilepsy (Barañano and Hartman 2008). Di Lorenzo et al. evaluated KD and standard low‐calorie dietary approaches (1200–1500 kcal/day) in patients with migraine headaches. The frequency of headaches and drug consumption was reduced in the KD with a 90% responder rate compared to a standard low‐calorie diet (Di Lorenzo et al. 2013). In another study, Di Lorenzo et al. investigated the clinical features of migraines following a very low‐calorie KD and a standard low‐calorie diet in overweight women with migraines. The ketogenic group experienced a significant decrease in attack frequency, headache days, and medication usage within the initial month of dietary intervention. Notably, a transient exacerbation of migraine symptoms was found after stopping the KD. Moreover, a standard low‐calorie diet could decrease the number of days with headaches and medication use after 3 months and the frequency of attacks after 6 months of diet prescription (Di Lorenzo et al. 2015). A prospective open‐label clinical trial indicated that the KD led to a significant decrease in the duration and frequency of migraine attacks, along with normalization at the cerebral cortex level after 1 month of intervention among individuals with migraine (Di Lorenzo, Coppola et al. 2019). In agreement with the literature, Bongiovanni et al. (2021) reported that the KD significantly reduced the duration and intensity of pain, days with symptoms, and medication use after 3 months of intervention. Additionally, in a randomized crossover clinical trial, a very low‐calorie KD was superior to a very low‐calorie non‐KD in the reduction of days with symptoms and migraine attacks, suggesting the KD may be a beneficial therapeutic approach for people with overweight and episodic migraine (Di Lorenzo, Pinto et al. 2019).

Overall, both observational and intervention studies have provided evidence in support of a promising intervention of the KD in the clinical characteristics of migraine headaches. More research is required to validate the effectiveness of the KD in treating migraines, to assess the optimal intervention duration, its feasibility in people with normal body weight, its utility in the pediatric population, and its relationship with the prevention of common migraines.

#### 2.2.5 Low‐Fat Diet

The KD has recently been proposed as a potential method to reduce the symptoms associated with migraine episodes. Before this, it was suggested that a diet high in fat might lead to headaches because the levels of plasma serotonin alter in association with increased platelet aggregation (Bic et al. 1998); therefore, a low‐fat diet may help prevent headaches (Bic et al. 1999). An evaluation conducted in 1999 by Bic and et al. focused on the impact of a low‐fat diet on the management of migraine headaches. A total of 54 patients were enrolled to reduce the consumption of dietary fats to less than 20 g/day for 3 months. The findings indicated that a low‐fat diet could significantly decrease headache frequency, intensity, and the requirement for medication use (Bic et al. 1999). Furthermore, a randomized crossover trial highlighted a notable decrease in headache intensity, frequency, and abortive medication usage in individuals adhering to a low‐fat vegan diet compared to those in the placebo group. The placebo group received a capsule containing alpha‐linolenic acid and vitamin E (Bunner et al. 2014). In 2015, a crossover trial was carried out to assess the role of a low‐lipid diet for acute migraine attack control. Subjects were instructed to receive a low‐lipid diet with < 20% total daily energy intake from fats for 12 weeks. The findings of the study revealed that people on a low‐fat diet had a remarkable decrease in both the frequency and severity of migraine attacks compared to those on a normal‐lipid diet (Ferrara et al. 2015). Although the number of studies investigating low‐fat diets is limited, it may be concluded that changes in the intake of dietary fats can influence migraine patterns, considering the effect of dietary fats on prostaglandin synthesis (Antonova et al. 2013). Prostaglandins, which are synthesized from essential fatty acids, play a role in the function of platelet and vascular tone regulation. These mediators also take part in the control of acute and chronic inflammation. For example, PGE1, made from linoleic acid (omega‐6), can lead to migraine headaches through its vasodilatory role. In contrast, omega‐3 fatty acids may improve migraine headache symptoms by decreasing platelet aggregation and affecting the pathway of serotonin biosynthesis. Therefore, the type and amount of dietary fats can influence inflammatory responses (Razeghi Jahromi et al. 2019). Moreover, several studies reported that a high‐fat diet increases low‐density lipoprotein‐cholesterol concentration and consequently elevates platelet aggregation. On the other hand, platelet accumulation through the secretion of serotonin and its vasoconstriction effects and the production of inflammatory markers, neuropeptides, and prostaglandins can be effective in causing migraine attacks. Thus, modulation in the type and amount of dietary fats seems to improve the clinical characteristics of migraine headaches (Borgdorff and Tangelder 2012).

#### 2.2.6 Low‐Glycemic Diet

A low‐glycemic diet is a dietary pattern that eliminates food items containing sugar, added sugar, and starchy carbohydrates to decrease the reliance on insulin for postprandial meal clearance. This pattern recommends consuming more carbohydrates from sources with a low glycemic index (60–80 g/day), such as lentils, whole grains, bran cereals, peas, and carrots (Lane et al. 2021). Some studies have reported the effectiveness of a low‐glycemic diet in treating neurological disorders, such as epilepsy and migraine (Evcili et al. 2018; Finsterer and Frank 2019; Neri et al. 2023). Evcili et al. assessed a low‐glycemic diet compared to standard prophylactic medications in patients with migraine. Both groups experienced a significant reduction in migraine attacks after one month of dietary control. While a low‐glycemic diet led to a remarkable reduction in headache intensity after 12 weeks. So, they concluded that consuming a diet with a low glycemic index can be a reliable and therapeutic method to decrease migraine attacks (Evcili et al. 2018). Moreover, an individualized low‐glycemic diet based on continuous glucose monitoring provided by a novel digital health application exhibited marked enhancements in subjects with more than one monthly migraine episode (Schröder et al. 2022). Consistently, Lelleck et al. undertook two prospective studies involving migraine patients utilizing a personalized low‐glycemic diet facilitated by a digital therapeutic platform over a 3‐month period. The participants reported a notable decline in migraine days by 2.40 days, HIT‐6 by 3.17 points, and MIDAS by 13.45 points (Lelleck et al. 2022). Overall, the exact mechanisms by which a low‐glycemic diet may decrease migraine symptoms are not fully known. A possible mechanism could be that insulin secretion increases after consuming fast‐absorbing carbohydrates in association with high postprandial glucose responses and subsequent declines in blood glucose levels below premeal levels. This is usually interpreted as a complex physiological counterregulatory reaction with excessive insulin secretion. One of the causes of hyperinsulinemia may be increased secretion of CGRP, which has an impact on plasma glucose levels and can cause relative hyperglycemia. Therefore, adhering to a low‐glycemic dietary pattern contributes to reduced postprandial glucose responses and enhanced insulin sensitivity (Schröder et al. 2022). Moreover, it is suggested that this approach may help to conserve the energy supply of the central nervous system, thereby offsetting potential energy deficits within the central nervous system during migraines. In addition, lower oxidative stress in low‐glycemic diets could be another explanatory mechanism for adherence (Ceriello et al. 2002). However, further research is imperative to provide necessary insights into the effects of low‐glycemic diets on migraine features.

#### 2.2.7 Gluten‐Free Diet

A gluten‐free diet entails refraining from the ingestion of barley, rye, malt, wheat, and their byproducts while including gluten‐free food items, like potatoes, corn, rice, and quinoa, as well as naturally gluten‐free food groups, including vegetables, fruits, nuts, seeds, seafood, legumes, meat, and dairy products (Moskatel and Zhang 2022). This diet is recommended for patients with celiac disease, leading to a remarkable improvement in digestive symptoms that can be seen within a few days to a few weeks after starting the diet. Moreover, studies have shown that exposure to or avoidance of certain foods can apply to the management of some disorders, such as migraine attacks (Martin and Vij 2016). This suggests a significant correlation between the clinical characteristics of migraines and dietary issues, such as intestinal dysfunction during migraine headaches, specific food sensitivities (gluten, histamine food sources, and dairy products), food triggers, and increased migraine attacks with obesity (Finkel et al. 2013). An observational study reported that the prevalence of migraine was approximately double in patients with celiac disease and irritable bowel syndrome compared to controls (Dimitrova et al. 2013). Therefore, gluten may be related to headache attacks in subjects with celiac or possibly in subjects with sensitivity to non‐celiac gluten. It is suggested that a gluten‐free diet may improve migraine/headache, which is one of the extra‐intestinal manifestations of celiac disease (Martin and Vij 2016). Beuthin et al. (2020) concluded that individuals diagnosed with celiac who also experience headaches or migraines are likely to have less severe and fewer headaches following a gluten‐free diet. Moreover, prospective observational studies reported that subjects with celiac disease and headaches exhibited a reduction in both the frequency and severity of headaches after initiating a gluten‐free diet (Ameghino et al. 2019). It's worth noting that following a gluten‐free diet may be related to weight gain due to higher consumption of carbohydrates, fats, and food items with a high glycemic index, which could potentially be harmful to migraine sufferers (Moskatel and Zhang 2022). So far, how a gluten‐free diet can improve migraine symptoms remains unclear. A possible explanation is that a gluten‐free diet improves intestinal inflammatory responses, reduces circulating inflammatory markers and antibody concentration, reduces nervous system hypersensitivity and vascular tone disturbances, and enhances intestinal absorption of nutrients (Arzani et al. 2020). However, more clinical trials are imperative to comprehensively elucidate the effects of a gluten‐free diet on migraine symptoms.

#### 2.2.8 Fasting Diet

Various fasting regimens have recently gained popularity as health‐promoting strategies that are attracting public interest (Mirrazavi and Behrouz 2024). In humans, fasting is delineated as the total and voluntary refraining from or intake of minimal quantities of sustenance and beverages for durations typically ranging from 12 h to 3 weeks. This practice has historical importance and exists in different forms and frequencies in almost all religions. Observably, Muslims engage in fasting from sunrise to sunset throughout the month of Ramadan annually, while adherents of Christianity, Judaism, Buddhism, and Hinduism traditionally observe fasting on specific days within the calendar year (Longo and Mattson 2014). Ramadan fasting has been reported as one of the dietary triggers of migraines because it may be associated with hypoglycemia, dehydration, and sleep disorders (Finocchi and Sivori 2012). Studies indicated that delaying or skipping a meal can exacerbate migraine episodes. Migraineurs may exhibit a diminished ability to deal with the consequences of hypoglycemia, rendering them more susceptible to the implications of fasting compared to those without migraine symptoms (Dalkara and Kılıç 2013). In this regard, a prospective cohort observational study demonstrated that Ramadan fasting led to an increase in the use of medications and the frequency of attacks in patients with migraines (Ragab et al. 2021). Consistent with other observational studies, a fasting diet is a trigger for migraine headache attacks (Abu‐Salameh et al. 2010; Al‐Shimmery 2010; Yadav et al. 2010). The mechanism of vulnerability to migraine attacks caused by meal omission or fasting is not fully understood. It seems that insulin‐induced hypoglycemia is not a suitable model to understand the mechanism of action of fasting regimens as triggers of migraines. Prolonged periods of hypoglycemia, lack of sympathetic activities or glucagon secretion in the early hours of fasting, and extended sympathetic activities during prolonged fasting can decrease glycogen‐derived glucose within peri‐synaptic astrocyte endfeet at the commencement of heightened synaptic activity. As a result, these events may cause an imbalance between inhibitory and excitatory nerve endings, leading to collective depolarization of neurons and astrocytes in a network and ultimately causing headaches (Dalkara and Kılıç 2013). It is recommended to undertake interventional investigations to scrutinize the impacts of diverse fasting diets on migraines.

## 3 Conclusion

This narrative review highlights research demonstrating the impact of various dietary patterns on the clinical features of migraine. Following certain patterns, such as the MedDiet, DASH, ketogenic, low‐fat diet with emphasis on consumption of n‐3 fatty acids, low‐glycemic diet, and gluten‐free diet, especially in gluten‐sensitive people, has shown promising results for improving migraine symptoms. Overall, these dietary strategies may exert a pivotal role in different cellular pathways, including improving mitochondrial function, neuroprotection, regulating vascular tone, inhibiting oxidative stress and neuroinflammation, and reducing levels of CGRP that are implicated in the pathophysiology of migraine. These results should stimulate further meticulously designed trials to optimize the efficacy of various dietary interventions for migraine management. Subsequent investigations should consider factors including optimal duration of dietary intervention, longer duration of follow‐ups, proper control group, migraine characteristics (with or without aura, chronic, episodic, presence of comorbidities), neurometabolic features, microbiome assessments, hormones, neurotransmitters, and adipocytokines levels.

## Author Contributions

**Vahideh Behrouz**: conceptualization, writing – original draft, writing – review and editing, supervision, methodology, investigation. **Elnaz Hakimi**: writing – original draft, methodology. **Elias Mir**: software.

## Conflicts of Interest

The authors declare no conflicts of interest.

## Peer Review

The peer review history for this article is available at https://publons.com/publon/10.1002/brb3.70652

## Data Availability

All original contributions featured in the research are incorporated within the article and supplementary materials. For additional information, inquiries can be directed to the corresponding author.
